# Endoscopic Biliary Darinage (EBD) versus Percutaneous Transhepatic Biliary Drainage (PTBD) for biliary drainage in patients with Perihilar Cholangiocarcinoma (PCCA): A systematic review and meta-analysis

**DOI:** 10.1016/j.clinsp.2022.100163

**Published:** 2023-01-19

**Authors:** Caroline Flaksbaum Moll, Diogo Turiani Hourneaux de Moura, Igor Braga Ribeiro, Igor Mendoça Proença, Epifanio Silvino do Monte Junior, Sergio A. Sánchez-Luna, Maria Fernanda Shinin Merchán, Josselyn Mariana Vera Intriago, Wanderley Marques Bernardo, Eduardo Guimarães Hourneaux de Moura

**Affiliations:** a Serviço de Endoscopia Gastrointestinal, Departamento de Gastroenterologia, Hospital das Clínicas, Faculdade de Medicina, Universidade de São Paulo (HCFMUSP), São Paulo, SP, Brazil; b Basil I. Hirschowitz Endoscopic Center of Excellence, Division of Gastroenterology and Hepatology, Department of Internal Medicine, The University of Alabama at Birmingham Heersink School of Medicine, Birmingham, Alabama, United States

**Keywords:** Endoscopic retrograde cholangiopancreatography, Percutaneous transhepatic biliary drainage, Cholangiocarcinoma, Klatskin, Biliary, Stent, Drainage, Cancer

## Abstract

Biliary drainage for Perihilar Cholangiocarcinoma (PCCA) can be performed either by endoscopic retrograde cholangiopancreatography or Percutaneous Transhepatic Biliary Drainage (PTBD). To date there is no consensus about which method is preferred. Taking that into account, the aim of this study is to compare Endoscopic Biliary Drainage (EBD) versus percutaneous transhepatic biliary drainage in patients with perihilar cholangiocarcinoma through a systematic review and metanalysis. A comprehensive search of multiple electronic databases was performed. Evaluated outcomes included technical success, clinical success, post drainage complications (cholangitis, pancreatitis, bleeding, and major complications), crossover, hospital length stay, and seeding metastases. Data extracted from the studies were used to calculate Mean Differences (MD). Seventeen studies were included, with a total of 2284 patients (EBD = 1239, PTBD = 1045). Considering resectable PCCA, the PTBD group demonstrated lower rates of crossover (RD = 0.29; 95% CI 0.07‒0.51; *p* = 0.009 I² = 90%), post-drainage complications (RD = 0.20; 95% CI 0.06‒0.33; *p* < 0.0001; I² = 78%), and post-drainage pancreatitis (RD = 0.10; 95% CI 0.05‒0.16; *p* < 0.0001; I² = 64%). The EBD group presented reduced length of hospital stay (RD = -2.89; 95% CI -3.35 ‒ -2,43; *p* < 0.00001; I² = 42%). Considering palliative PCCA, the PTBD group demonstrated a higher clinical success (RD = -0.19; 95% CI -0.27 ‒ -0.11; *p* < 0.00001; I² = 0%) and less post-drainage cholangitis (RD = 0.08; 95% CI 0.01‒0.15; *p* = 0.02; I² = 48%) when compared to the EBD group. There was no statistical difference between the groups regarding: technical success, post-drainage bleeding, major post-drainage complications, and seeding metastases.

## Highlights

• There is no consensus about which method is preferred for biliary drainage for Perihilar Cholangiocarcinoma (PCCA).
• Endoscopic retrograde cholangiopancreatography (EBD) or Percutaneous Transhepatic Biliary Drainage (PTBD) are options of choice.
• PTBD is superior to EBD regarding crossover rate, overall post-drainage complications, and post-drainage pancreatitis.
• EBD is superior to PTBD when it comes to hospital length of stay.
• For the palliation of PCCA, PTBD is superior to EBD in terms of clinical success and post-drainage cholangitis.

## Introduction

Cholangiocarcinoma can involve the intra and extrahepatic bile ducts.^1^ Tumor growth can lead to obstructive jaundice, which may require biliary drainage either in a preoperative or palliative context. It is also related to a high mortality rate and is typically associated with a less than 50% survival in 5 years even after surgical resection in stages III‒IV.^2^

Surgical treatment of Perihilar Cholangiocarcinoma (PCCA) may involve right hepatectomy, left hepatectomy, extended right hepatectomy or extended left hepatectomy and the strategy choice requires evaluation of lobar atrophy and extend of biliary disease.^3^ Segment I resection is always indicated due to risk of tumor involvment.^3^. Such large liver resections are associated with up to 18% mortality rate in cholestatic patients.^4^ Therefore, in selected patients, pre-operative biliary drainage is indicated to imporve liver function, alleviate jaundice and promote regenaration of the remnant liver.^4^ Nevertheless, preoperative drainage has also been related to worse postoperative outcomes in patients with predicted future liver remnant of ≥ 30%, which is probably associated with biliary instrumentation, leading to bacterial contamination and, finally, to cholangitis.^5^ Facing that, whether to perform preoperative biliary drainage remains controversial, and the most recent European Society of Gastrointestinal Endoscopy (ESGE) guideline from 2017 suggests drainage for patients with cholangitis or future remnant liver volume of ≤ 30% after surgery.^6^

Regarding unresectable cholangiocarcinoma, mostly all patients undergo biliary drainage to palliate pruritus, weakness, and to improve nutritional status.^7^ Biliary drainage is also associated with increased survival in this population.^8^^,^^9^

When it comes to choosing between Endoscopic Biliary Drainage (EBD) or Percutaneous Transhepatic Biliary Drainage (PTBD) in Perihilar Cholangiocarcinoma (PCCA), there is not enough data to establish a consensus, and in each case, several aspects are taken into account before making this decision, such as anatomic localization of the obstruction, device availability in the referred medical center, local expertise, preoperative or palliative purposes, and patient status and preference.^10^

Therefore, this systematic review and meta-analysis aim to elucidate which is the optimal approach for biliary drainage in patients with resectable and palliative PPCA, based on the available literature.

## Materials and methods

### Protocol and registration

The study protocol was registered in the International Prospective Register of Systematic Reviews (PROSPERO) under the file number CRD42021256738 and was approved by the Ethics Committee of Hospital das Clínicas, Faculty of Medicine at The University of São Paulo. This systematic review and meta-analysis were performed in conformity with the recommendations from the Cochrane Handbook of Systematic Reviews of Interventions and the Preferred Reporting Items for Systematic Reviews and Meta-analysis guidelines (PRISMA).^11^

### Eligibility criteria

The eligibility criteria were cohort studies or Randomized Controlled Trials (RCTs) comparing EBD versus PTBD for patients with PCCA requiring biliary drainage. The exclusion criteria were studies that included other etiologies of obstructive jaundice, and studies that only included distal cholangiocarcinoma or did not specify the anatomic location of the tumor.

### Search strategy and information sources

Individualized searches of multiple electronic databases (MEDLINE, Embase, Cochrane, LILACS, and gray literature) were performed based upon a standardized protocol from their inception through February 2022. Data search was made without language or publication date limitations. The following search strategy was used in all databases: (Cholangiocarcinomas OR Cholangiocarcinoma OR Cholangiocellular Carcinoma OR Cholangiocellular Carcinomas OR Tumor, Klatskin OR Klatskin's Tumor) AND (stent OR stents OR drainage OR endoscopy OR endoscopic OR percutaneous).

### Study selection and data extraction

Two researchers independently conducted the eligibility screening. From the initial search results, duplicate articles were excluded, and the titles and abstracts of all potentially relevant studies were screened for eligibility. Any disagreements were settled by consensus or by consulting a third reviewer.

An Excel spreadsheet was used to organize relevant data extracted from the selected articles, which consisted of the name of the first author, year of publication, type of study, the total number of patients, number of patients submitted to PTBD, number of patients submitted to EBD, population (resectable cholangiocarcinoma vs. palliative patients), Bismuth-Corlette classification, and outcomes. The evaluated outcomes were technical success, clinical success, post drainage complications (cholangitis, pancreatitis, bleeding, and major complications), crossover, hospital length stay, and seeding metastases.

### Risk of bias in individual studies and quality of evidence

The risk of bias was assessed by Cochrane's Risk of Bias in Non-randomized Studies of Interventions (ROBINS-I), a tool for evaluating the risk of bias in non-randomized studies.^12^

The quality of evidence was assessed utilizing the objective criteria from Grading Recommendations Assessment, Development, and Evaluation (GRADE) for each of the pre-specified results and outcomes using the GRADEpro ‒ Guideline Development Tool software (McMaster University, 2015; Evidence Prime, Inc., Ontario, Canada).^13^

### Statistical analysis

The data extracted from the outcomes of interest were meta-analyzed using the RevMan software (Review Manager Software version 5.4 ‒ Cochrane Collaboration Copyright© 2020).

For dichotomous variables, risk difference was used, through the Mantel Haenszel test, with a 95% Confidence Interval (95% CI).

For continuous variables, Mean Difference (MD) was used, and it was calculated through the inverse variance. When standard deviation was not available in the article, it was estimated through the Hozo test.^14^

Heterogeneity was calculated using the Higgins test (I²). When heterogeneity was < 50%, a fixed effect was used and when it was > 50%, a random effect was applied.^14^ Values of *p* < 0.05 were considered statistically significant.

## Results

### Search results and study characteristics

**Fig. 1:**
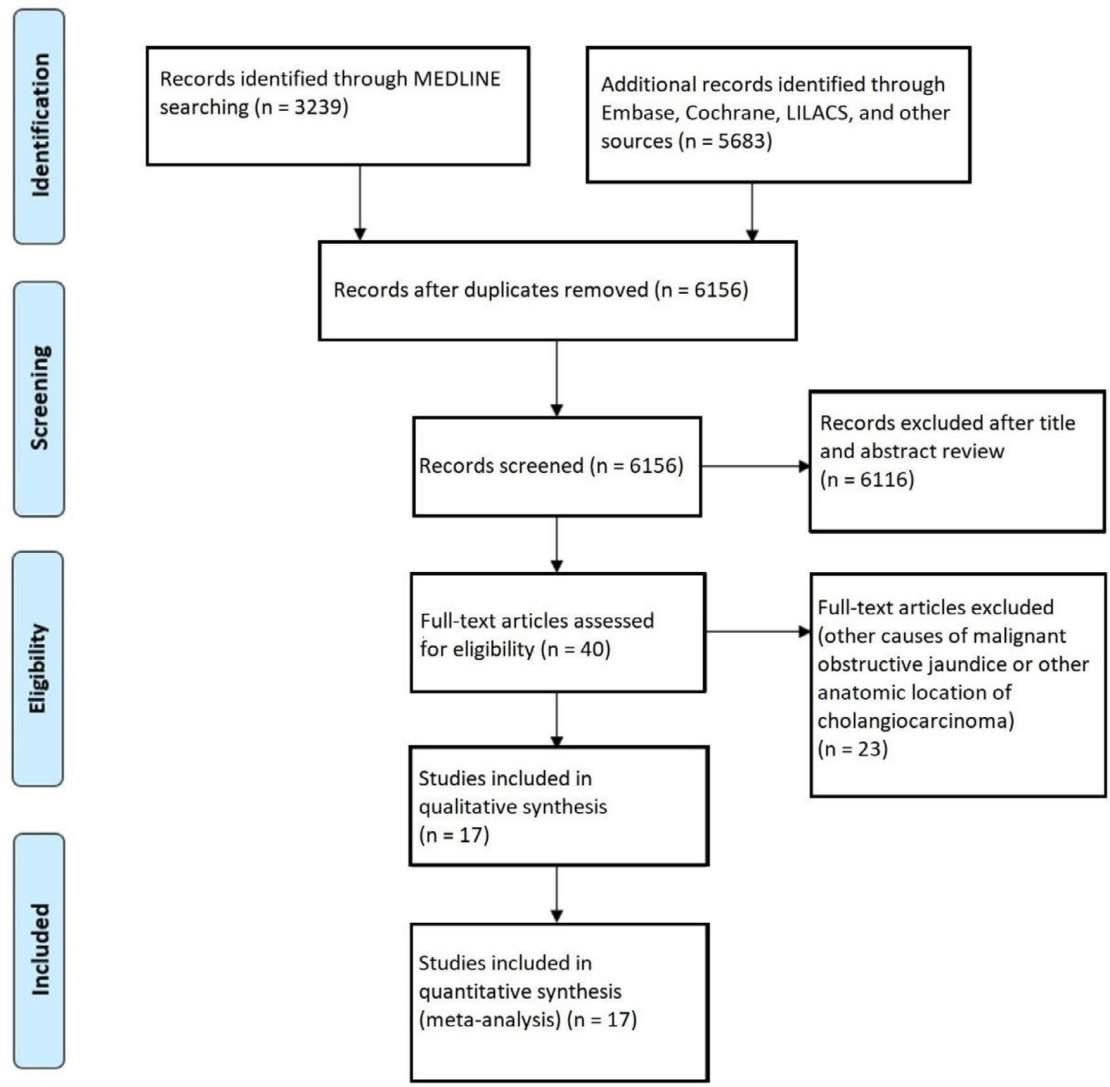
Flow diagram showing study selection process.

The initial search identified a total of 3239 studies. After removal of duplicates, evaluation of titles and abstracts, and full-text analysis, 16 retrospective cohort studies^15^, ^16^, ^17^, ^18^, ^19^, ^20^, ^21^, ^22^, ^23^, ^24^, ^25^, ^26^, ^27^, ^28^, ^29^, ^30^ and one Randomized Controlled Trial (RCT)^4^ were included with a total of 2284 patients (EBD = 1239, PTBD = 1045) (Fig. 1). Table 1 summarizes the characteristics of the included studies.

**Table 1:** Characteristics of included studies.

Author	Publication year	Study design	Population	Patients (n)	Bismuth- Corlette	Intervention / Control	Technical success	Clinical success	Post-drainage complications
Ba Y	2020	Cohort	Resectable cholangiocarcinoma	180	II/III/IV	99 EBD	‒	‒	74/99
						81 PTBD	‒	‒	27/81
Born P	2000	Cohort	Unresectable cholangiocarcinoma	59	II/III/IV	20 EBD	‒	‒	6/20
						39 PTBD	‒	‒	13/39
Coelen RJS	2018	RCT	Resectable cholangiocarcinoma	54	I/II/III/IV	27 EBD	20/27	17/27	18/27
						27 PTBD	25/27	21/27	17/27
Hirano S	2014	Cohort	Resectable cholangiocarcinoma	141	I/II/IIIa/IIIb/IV	74 EBD	‒	‒	12/74
						67 PTBD	‒	‒	14/67
Jo JH	2016	Cohort	Resectable cholangiocarcinoma	98	I/II/IIIa/IIIb/IV	55 EBD	55/61	‒	20/55
						43 PTBD	36/37	‒	12/43
Kawakami H	2011	Cohort	Resectable cholangiocarcinoma	128	I/II/IIIa/IIIb/IV	80 EBD	‒	‒	36/80
						48 PTBD	‒	‒	15/48
Kim KM	2015	Cohort	Resectable cholangiocarcinoma	106	I/II/III/IV	44 EBD	25/44	‒	24/44
						62 PTBD	36/62	‒	14/62
Kloek JJ	2009	Cohort	Resectable cholangiocarcinoma	101	I/II/III/IV	90 EBD	73/90	‒	73/90
						11 PTBD	11/11	‒	4/11
Komaya K	2016	Cohort	Resectable cholangiocarcinoma	320	I/II/III/IV	152 EBD	‒	‒	‒
						168 PTBD	‒	‒	‒
Lee SH	2007	Cohort	Unresectable cholangiocarcinoma	134	II/III/IV	34 EBD	‒	27/34	13/34
						100 PTBD	‒	95/100	30/100
Liang X	2021	Cohort	Unresectable cholangiocarcinoma	145	I/II/III/IV	97 EBD	172/173	50/95	38/97
						48 PTBD	97/97	31/46	19/48
Paik WH	2008	Cohort	Unresectable cholangiocarcinoma	85	III/IV	44 EBD	‒	34/44	13/44
						41 PTBD	‒	38/41	13/41
Walter T	2012	Cohort	Resectable + Unresectable cholangiocarcinoma	129	NA/I/II/III/IV	87 EBD	68/87	43/87	23/87
						42 PTBD	41/42	33/42	11/42
Wiggers JK	2015	Cohort	Resectable cholangiocarcinoma	245	I/II/IIIa/IIIb/IV	157 EBD	‒	‒	‒
						88 PTBD	‒	‒	‒
Zhang XF	2017	Cohort	Resectable cholangiocarcinoma	196	I/II/III/IV	92 EBD	75/92	‒	‒
						104 PTBD	90/104	‒	‒
Zheng R	2019	Cohort	Resectable cholangiocarcinoma	81	‒	45 EBD	45/45	45/45	16/45
						36 PTBD	36/36	29/36	4/36
Zhu J	2020	Cohort	Unresectable cholangiocarcinoma	82	I/II/III/IV	42 EBD	‒	‒	26/42
						40 PTBD	‒	‒	8/40

Two studies^14^^,^^18^ divided the EBD population from those who underwent Endoscopic Nasobiliary Drainage (ENBD). For this meta-analysis, the two groups were merged into the EBD group. No distinction was made between plastic and metal stents neither in the EBD group or the PTBD group due to lack of uniformity in the data provided by the studies. Only seven studies specified which stent material was used.^15^^,^^16^^,^^23^, ^24^, ^25^, ^26^^,^^30^ From those, one used only plastic stents in both groups,^15^ two only used metallic stents in both groups,^25^^,^^30^ two used plastic and metal stents in the EBD group and only plastic stents in the PTBD group,^24^^,^^26^ and, finally, two used both types of stents in EBD and PTBD patients.^16^^,^^23^

There was also no distinction regarding the Bismuth-Corlette classification.^31^ Some studies included patients with Bismuth from I to IV,^4^^,^^17^, ^18^, ^19^, ^20^, ^21^, ^22^^,^^26^, ^27^, ^28^^,^^30^ some included II to IV,^15^^,^^16^^,^^23^^,^^24^ one study included only III and IV^25^ and the other remaining studies did not specify the patients’ Bismuth-Corlette classification.^29^

Two separate meta-analyses were performed, one for the outcomes regarding the treatment of resectable PCCA and another one for the palliation of PCCA.

### Risk of bias and quality of evidence assessment

**Fig. 2:**
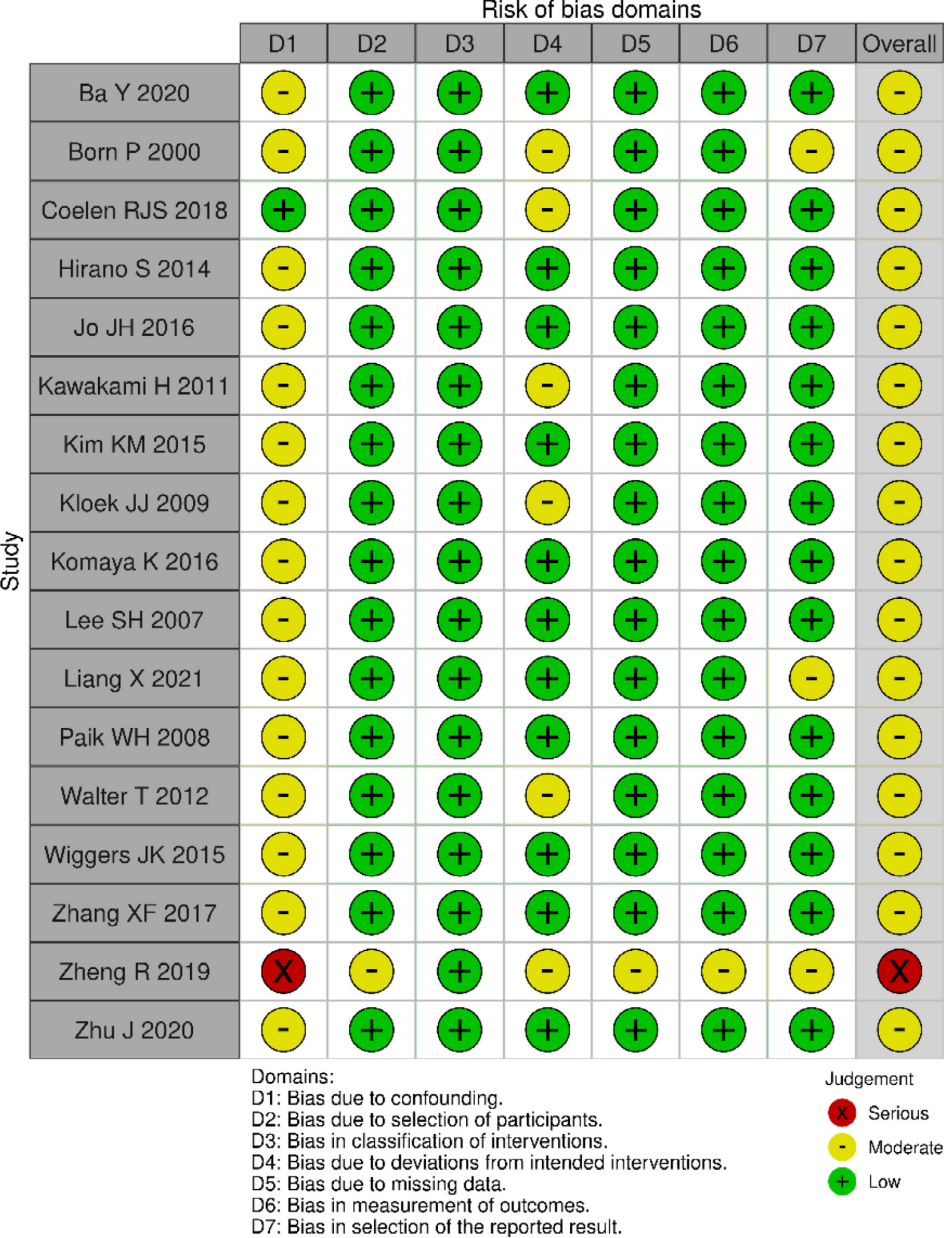
Risk of bias for ROBINS-I.

The risk of bias assessed by the ROBINS-I tool is shown in Fig. 2. Sixteen^4^^,^^15^, ^16^, ^17^, ^18^, ^19^, ^20^, ^21^, ^22^, ^23^, ^24^, ^25^, ^26^, ^27^, ^28^^,^^30^ out of the selected studies presented a moderate overall risk of bias and one study^29^ presented a serious risk of bias.

All the evaluated outcomes presented a very low level of evidence according to the GRADE for resectable PCCA (Supplementary Material, Appendix 1) and also for palliative PCCA (Supplementary Material, Appendix 2).

## Meta-analysis

### Resectable PCCA-related outcomes

#### Technical success in resectable PCCA

**Fig. 3:**
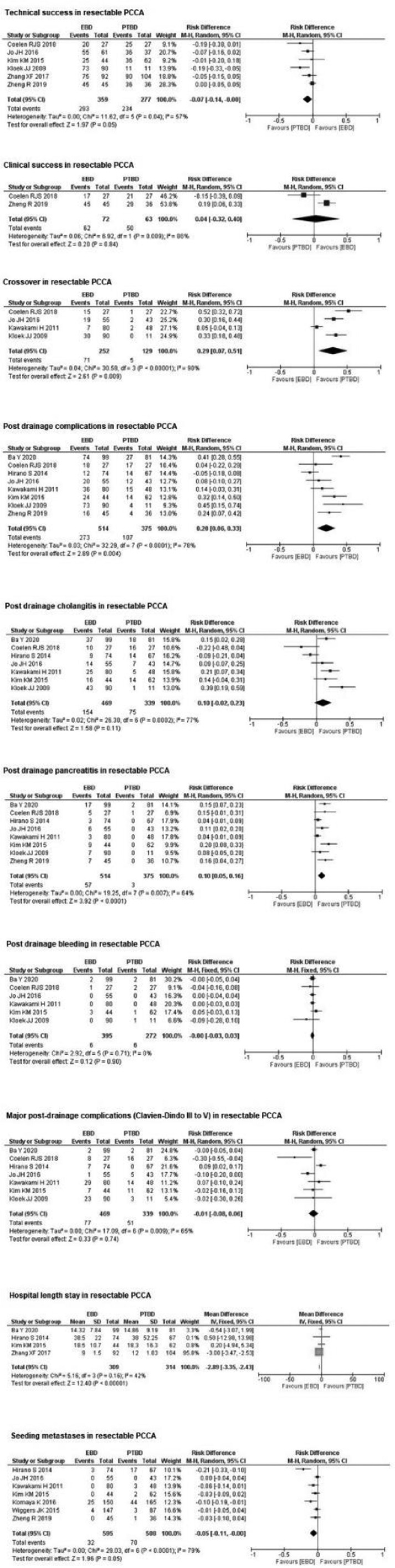
Forrest Plots for resectable PCCA.

Six studies,^4^^,^^18^^,^^20^^,^^21^^,^^28^^,^^29^ with a total of 636 patients (359 in the EBD group and 277 in the PTBD group) evaluated technical success in resectable PCCA. There was no statistically significant difference between the two groups (RD = −0.07; 95% CI −0.14‒0.00; *p* = 0.05; I² = 57%) (Fig. 3).

#### Clinical success in resectable PCCA

Two studies,^4^^,^^29^ with a total of 135 patients (72 in the EBD group and 63 in the PTBD group) evaluated clinical success in resectable PCCA. There was no statistically significant difference between the two groups (RD = 0.04; 95% CI −0.32‒0.40; *p* = 0.84; I² = 86%) (Fig. 3).

#### Crossover in resectable PCCA

Four studies,^4^^,^^18^^,^^19^^,^^21^ with a total of 381 patients (252 in the EBD group and 129 in the PTBD group) evaluated crossover in resectable PCCA. In the PTBD group, there was a 29% decrease in crossover (RD = 0.29; 95% CI 0.07‒0.51; *p* = 0.009 I² = 90%) compared to the EBD group (Fig. 3).

#### Post drainage complications in resectable PCCA

Eight studies,^4^^,^^15^^,^^17^, ^18^, ^19^, ^20^, ^21^^,^^29^ with a total of 889 patients (514 in the EBD group and 375 in the PTBD group) evaluated post-drainage complications in resectable PCCA. In the PTBD group, there was a 20% reduction in post-drainage complications compared to the EBD group (RD = 0.20; 95% CI 0.06‒0.33; *p* < 0.0001; I² = 78%) (Fig. 3).

#### Post drainage cholangitis in resectable PCCA

Seven studies,^4^^,^^15^^,^^17^, ^18^, ^19^, ^20^, ^21^ with a total of 808 patients (469 in the EBD group and 339 in the PTBD group) evaluated post-drainage cholangitis in resectable PCCA. There was no statistically significant difference between the two groups (RD = 0.10; 95% CI −0.02‒0.23; *p* = 0.11; I² = 77%) (Fig. 3).

#### Post drainage pancreatitis in resectable PCCA

Eight studies,^4^^,^^15^^,^^17^, ^18^, ^19^, ^20^, ^21^^,^^29^ with a total of 889 patients (514 in the EBD group and 375 in the PTBD group) evaluated post-drainage pancreatitis in resectable PCCA. In the PTBD group, there was an 10% reduction in post-drainage pancreatitis compared to the EBD group (RD = 0.10; 95% CI 0.05‒0.16; *p* < 0.0001; I² = 64%) (Fig. 3).

#### Post drainage bleeding in resectable PCCA

Six studies,^4^^,^^15^^,^^18^, ^19^, ^20^, ^21^ with a total of 667 patients (395 in the EBD group and 272 in the PTBD group) evaluated post-drainage bleeding in resectable PCCA. There was no statistically significant difference between the two groups (RD = 0.00; 95% CI −0.03‒0.03; *p* = 0.90; I² = 0%) (Fig. 3).

#### Major post-drainage complications (Clavien-Dindo III to V) in resectable PCCA

Seven studies,^4^^,^^15^^,^^17^, ^18^, ^19^, ^20^, ^21^ with a total of 808 patients (469 in the EBD group and 339 in the PTBD group) evaluated major post-drainage complications (Clavien-Dindo III to V) in resectable PCCA. There was no statistically significant difference between the two groups (RD = −0.01; 95% CI −0.08‒0.06; *p* = 0.74; I² = 65%) (Fig. 3).

#### Length of hospital stay in resectable PCCA

Four studies,^15^^,^^17^^,^^20^^,^^28^ with a total of 623 patients (309 in the EBD group and 314 in the PTBD group) evaluated the length of hospital stay in resectable PCCA. The EBD group showed a decrease in the number of days in hospital stay length when compared to the PTBD group (RD = −2.89; 95% CI −3.35 ‒ −2,43; *p* < 0.00001; I² = 42%) (Fig. 3).

#### Seeding metastases in resectable PCCA

Seven studies,^17^, ^18^, ^19^, ^20^^,^^22^^,^^27^^,^^29^ with a total of 1103 patients (595 in the EBD group and 508 in the PTBD group) evaluated seeding metastases in resectable PCCA. There was no statistically significant difference between the two groups (RD = −0.05; 95% CI −0.11‒0.00; *p* < 0.0001; I² = 79%) (Fig. 3).

### Palliation of PCCA-related outcomes

#### Technical success in palliative PCCA

**Fig. 4:**
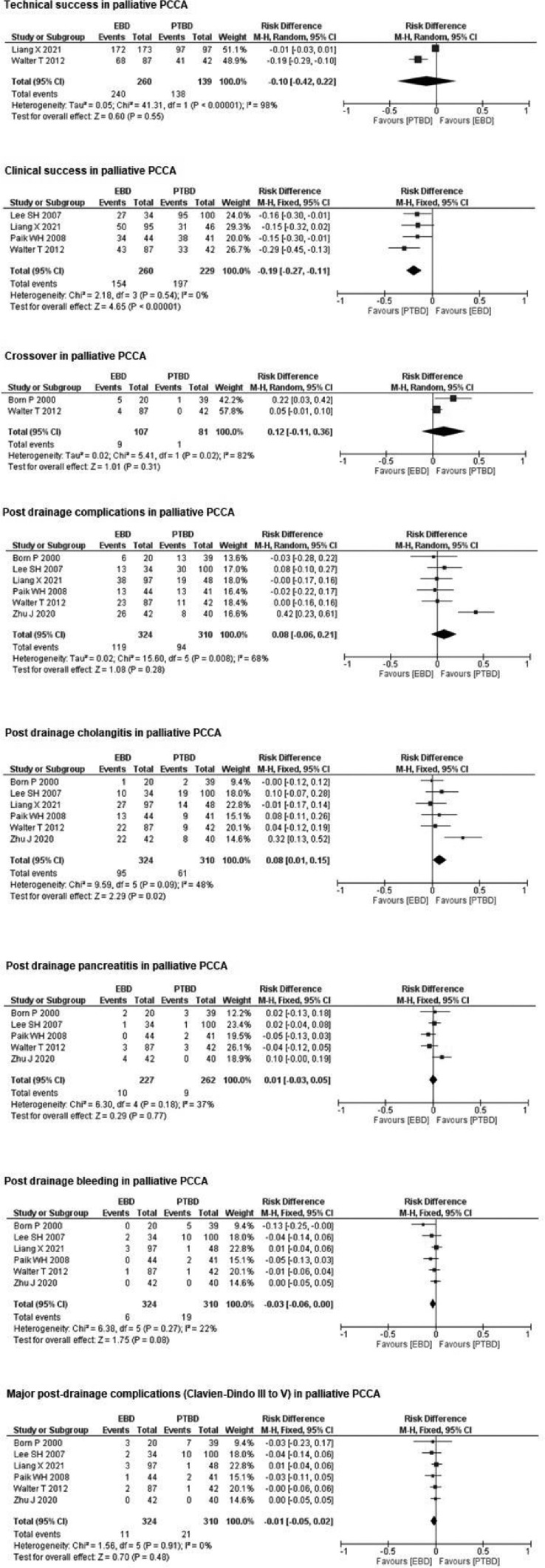
Forrest Plots for palliative PCCA.

Two studies,^24^^,^^26^ with a total of 399 patients (260 in the EBD group and 139 in the PTBD group) evaluated technical success in palliative PCCA. There was no statistically significant difference between the two groups (RD = −0.10; 95% CI −0.42‒0.22; *p* = 0.55; I² = 98%) (Fig. 4).

#### Clinical success in palliative PCCA

Four studies,^23^, ^24^, ^25^, ^26^ with a total of 489 patients (260 in the EBD group and 229 in the PTBD group) evaluated clinical success in palliative PCCA. In the PTBD group, there was a 19% increase in clinical success (RD = −0.19; 95% CI −0.27 ‒ −0.11; *p* < 0.00001; I² = 0%) compared to the EBD group (Fig. 4).

#### Crossover in palliative PCCA

Two studies,^16^^,^^26^ with a total of 188 patients (107 in the EBD group and 81 in the PTBD group) evaluated crossover in palliative PCCA. There was no statistically significant difference between the two groups (*R* = 0.12; 95% CI −0.11‒0.36; *p* = 0.31; I² = 82%) (Fig. 4).

#### Post drainage complications in palliative PCCA

Six studies,^16^^,^^23^, ^24^, ^25^, ^26^^,^^30^ with a total of 634 patients (324 in the EBD group and 310 in the PTBD group) evaluated post-drainage complications in palliative PCCA. There was no statistically significant difference between the two groups (RD = 0.08; 95% CI −0.06‒0.21; *p* = 0.28; I² = 68%) (Fig. 4).

#### Post drainage cholangitis in palliative PCCA

Six studies,^16^^,^^23^, ^24^, ^25^, ^26^^,^^30^ with a total of 634 patients (324 in the EBD group and 310 in the PTBD group) evaluated post-drainage cholangitis in palliative PCCA. In the PTBD group, there was an 8% reduction in post-drainage cholangitis compared to the EBD group (RD = 0.08; 95% CI 0.01‒0.15; *p* = 0.02; I² = 48%) (Fig. 4).

#### Post drainage pancreatitis in palliative PCCA

Five studies,^16^^,^^23^^,^^25^^,^^26^^,^^30^ with a total of 489 patients (227 in the EBD group and 262 in the PTBD group) evaluated post-drainage pancreatitis in palliative PCCA. There was no statistically significant difference between the two groups (RD = 0.01; 95% CI −0.03‒0.05; *p* = 0.77; I² = 37%) (Fig. 4).

#### Post drainage bleeding in palliative PCCA

Six studies,^16^^,^^23^, ^24^, ^25^, ^26^^,^^30^ with a total of 643 patients (324 in the EBD group and 310 in the PTBD group) evaluated post-drainage bleeding in palliative PCCA. There was no statistically significant difference between the two groups (RD = −0.03; 95% CI −0.06‒0.00; *p* = 0.08; I² = 22%) (Fig. 4).

#### Major post-drainage complications (Clavien-Dindo III to V) in palliative PCCA

Six studies,^16^^,^^23^, ^24^, ^25^, ^26^^,^^30^ with a total of 634 patients (324 in the EBD group and 310 in the PTBD group) evaluated major post-drainage complications (Clavien-Dindo III to V) in palliative PCCA. There was no statistically significant difference between the two groups (RD = −0.01; 95% CI −0.05‒0.02; *p* = 0.48; I² = 0%) (Fig. 4).

## Discussion

To the best of our knowledge, this systematic review and meta-analysis are the first to compare the outcomes of EBD and PTBD in both resectable and palliative PCCA. Furthermore, it has a larger number of patients, analyses, and a vaster scope of outcomes when compared to the other previous meta-analyses.^32^, ^33^, ^34^, ^35^

Regarding resectable PCCA, the results of this meta-analysis partially match the ones seen in other meta-analyses,^32^, ^33^, ^34^, ^35^ including lower rates of post-drainage complications in the PTBD group,^32^^,^^34^^,^^35^ higher post-drainage pancreatitis in the EBD group,^32^^,^^34^ and a higher rate of crossover in the EBD group.^32^ However, different results were found in this updated meta-analysis, including similar post-drainage cholangitis and a decrease in the length of hospital stay in the EBD group.

The higher rate of post-drainage complications and higher crossover rates seen in EBD are most likely associated with the manipulation of the papilla (i.e., post-ERCP pancreatitis) and challenging cannulation due to the thin distal bile duct. Even so, nowadays evidence shows^36^ that post-ERCP pancreatitis rates can be reduced by the use of rectal Non-Steroidal Anti-Inflammatory Drugs (NSAIDs), intravenous fluids, and pancreatic stent placement when the main pancreatic duct is inadvertently cannulated. The included studies did not specify if such measures were taken in the EBD group of patients, which may also interfere with our results.

One conflicting outcome was a lower rate of post-drainage cholangitis in the PTBD group by Hajibandeh S et al.,^32^ which was not seen in our study. The present meta-analysis included three additional studies,^4^^,^^15^^,^^17^ two retrospective,^15^^,^^17^ and one RCT.^4^ These results are expected since both endoscopic and radiological stents are usually effective in promoting drainage after adequate placement.

Furthermore, another different outcome when compared to the previous meta-analysis was a decrease in the length of hospital stay in the EBD group when compared to the PTBD group. Liu et al.^35^ did not find a difference between groups in their study. In our meta-analysis, we included one study that was not included in the previous meta-analysis.^28^ The reason for the exclusion was not mentioned. In that article, patients who underwent PTBD initially presented with slightly higher median levels of peak bilirubin when compared to patients who underwent EBD. It is possible that this could have led to a bias affecting that study's outcomes and consequently, the present study's analysis.

Also, the lack of statistical difference between EBD and PTBD in terms of seeding metastases is surprising. Previous literature demonstrated a higher rate of seeding metastases in the PTBD group.^32^ One hypothesis for that is that the definition of seeding metastases differs among studies. For instance, Komaya K et al.^22^ included in this group any peritoneal dissemination. Such a broad definition may include erroneously some patients that simply had disease progression, that would have led to peritoneal implantation/carcinomatosis regardless of the type of drainage performed.

In terms of resectable PCCA management, the ESGE consensus guidelines from 2017^6^ recommend against routine biliary drainage. In cases when drainage is required (cholangitis, the necessity of portal vein embolization, etc.), there is no definition of which modality of biliary drainage these patients should undergo and, even less, the correlation of that with the Bismuth-Corlette classification (i.e., optimal route of drainage depending on which type of Bismuth stricture is present). Considering safety profile, similar to our data, this consensus^6^ points out that most studies reported more adverse events related to EBD than PTBD. However, one large retrospective study^37^ showed that PTBD is associated with higher major post-hepatectomy morbidity (Clavien-Dindo III to V). This study^37^ was not included in our meta-analysis because it also included patients with gallbladder cancer. Furthermore, the only RCT^4^ included in this meta-analysis was prematurely closed due to a higher rate of mortality in the PTBD group. After its first annual report, it was seen that the PTBD group showed a statistically significantly higher mortality rate (RR = 3.67, 95% CI 1.15–11.69; *p* = 0.03). In the PTBD group, 3 patients died after biliary drainage and 8 died after surgical resection versus 3 post-drainage deaths in the EBD group. A possible explanation is that bile loss provoked by PTBD could lead to immunity impairment and a worse post-resection regenerative response. Nevertheless, the small number of patients included in the study (27 in each group) could have led to a type-I error and, futhermore, from the PTBD patients who died after surgical ressection, 5 died from postsurgical complications, 2 from myocardial infarction and 1 from progression of disease. Therefore, the results should be interpreted with caution, since these complications do not seem to be directly related to biliary drainage consequences.

Regarding nonresectable PCCA, our results are similar to a previous meta-analysis^33^ in terms of clinical success, showing a benefit for the PTBD group. However, our study demonstrated a lower number of episodes of cholangitis in the patients undergoing PTBD. Despite EBD having similar technical success to PTBD, it has a lower clinical success. This could be explained by stent malfunction (migration or obstruction) posterior to successful biliary drainage. Since the percutaneous drainage catheter is (at least in the first moment) external and sutured to the skin, it has a smaller likelihood of getting dislocated. It has also lesser chances of obstruction due to the larger diameter of some stents (up to 14 Fr). The higher number of cholangitis episodes in the EBD group may be related to ascending bacterial colonization of the bile due to the duodenal reflux of intestinal contents. Additionally, the use of uncovered Self-Expandable Metal Stents (u-SEMS) may also increase the rate of repeated cholangitis, as these stents can be cleaned off debris with balloon sweeps during subsequent ERCPs with the caveat of causing upstream ascending colonization of bacteria, but never removed. This may be the reason for more post-drainage cholangitis for nonresectable PCCA compared to resectable PCCA drainage.

According to the ESGE 2017 consensus guidelines,^6^ palliative drainage of PCCA from Bismuth-Corlette's I‒II should be performed by EBD and from III‒IV by either PTBD alone or PTBD combined with EBD. Unfortunately, all studies did not provide enough data to perform a correlated analysis based on the Bismuth-Corlette classification and biliary drainage technique.

Although our meta-analysis includes a higher number of studies and patients and is the first to analyze both patients who underwent pre-operative and palliative biliary drainage, our study is not exempt from limitations. The main limitation is that most of the used data come from retrospective cohort studies, which leads to a moderate risk of bias and a very low level of evidence in all evaluated outcomes. Another limitation, especially in regard to nonresectable PCCA is the impossibility to subdivide the data according to the Bismuth-Corlette classification. Bismuth I‒II patients may present better outcomes for EBD due to its anatomic location being easier accessed through this method, whereas Bismuth III‒IV patients may perform better with PTBD. Furthermore, the analysis included both plastic and metal stents, and an individual analysis could not be performed due to the lack of uniformity in the data provided by the articles.

Overall, PTBD presented with better outcomes than EBD in both resectable and palliative PCCA. Our study did not perform an analysis comparing the quality of life in both groups. Nevertheless, we believe that having internal drainage with EBD in lieu of an external one provided by a PTBD may be more comfortable for the patient as shown in studies that have compared ultrasound-guided gallbladder drainage (i.e., internal drainage) versus percutaneous cholecystostomy tube for the management of acute cholecystitis in non-surgical candidates.^38^

Furthermore, EBD performed similarly to PTBD in most evaluated outcomes. Therefore, we believe that EBD's advantages could outweigh its disadvantages and should be considered. Thus, the optimal drainage technique to choose in PCCA should be assessed with caution and we recommend an individualized approach, with consideration towards anatomy, personal, and local expertise, resources availability, and patient preferences. Further, RCTs are warranted to compare EBD versus PTBD with the hopes of clarifying which drainage modality may better serve the degree and site of obstruction in patients in PCCA.

## Conclusion

In terms of biliary drainage for resectable PCCA, PTBD is superior to EBD regarding crossover rate, overall post-drainage complications, and post-drainage pancreatitis, whereas EBD is superior to PTBD when it comes to hospital length of stay. For the palliation of PCCA, PTBD is superior to EBD in terms of clinical success and post-drainage cholangitis.

## Authors’ contributions

Moll, CF: Acquisition of data, analysis, interpretation of data, drafting the article, revising the article, final approval; Ribeiro IB, do Monte ES and Proença IM: Interpretation of data, revising the article, final approval; Ribeiro IB and de Moura DTH: Revising the article, final approval; Merchán MFS and Intriago JMV: Interpretation of data, revising the article, final approval; Sánchez-Luna SA: Drafting the article, revising the article, final approval; Bernardo WM: Analysis and interpretation of data, revising the article, final approval; de Moura EGH: Conception and design of the study, critical revision, final approval.

## Ethical statement

The study was approved by the Research Ethics Committee of the University of São Paulo School of Medicine Hospital das Clínicas. For this type of study formal consent is not required.

## Declaration of Competing Interest

DTHM: BariaTek ‒ Advisory Board Member (Consulting fees).

SASL: Recipient of the 2021 American Society for Gastrointestinal Endoscopy (ASGE) Endoscopic Training Award by the ASGE and Fujifilm. EGHM: Olympus ‒ Consultant (Consulting fees), Boston Scientific ‒ Consultant (Consulting fees).

## Appendix Supplementary materials

Image, application 1
