# Supplementary material for: Endoscopic Biliary Darinage (EBD) versus Percutaneous Transhepatic Biliary Drainage (PTBD) for biliary drainage in patients with Perihilar Cholangiocarcinoma (PCCA): A systematic review and meta-analysis
Source: Clinics (Sao Paulo). 2023 Jan 19;78:100163. doi: 10.1016/j.clinsp.2022.100163 (PMC10757298; doi:10.1016/j.clinsp.2022.100163)
Supplement: Supplementary file 1 [file mmc1.docx]

**CLINICS-D-22-00280_Supplementary Material**

**Supplementary Material** ‒ **Appendix 1** Quality of evidence by GRADE for resectable PCCA.

| **Certainty assessment** | | | | | | | **Nº of patients** | | **Effect** | | **Certainty** |
| --- | --- | --- | --- | --- | --- | --- | --- | --- | --- | --- | --- |
| **Nº of studies** | **Study design** | **Risk of bias** | **Inconsistency** | **Indirectness** | **Imprecision** | **Other considerations** | **PTBD** | **EBD** | **Relative (95% CI)** | **Absolute (95% CI)** |  |
| **Seeding metastases** | | | | | | | | | | | |
| 7 | Observational studies | Very serious^a^ | Very serious^b^ | Not serious | Serious^e^ | Publication bias strongly suspected^d^ | 70/508 (13.8%) | 32/595 (5.4%) | Not estimable | **50 more per 1.000** (from 0 fewer to 110 more) | ⨁◯◯◯ Very low |
| **Hospital length stay (days)** | | | | | | | | | | | |
| 4 | Observational studies | Serious^a^ | Not serious | Not serious | Very serious^g^ | Publication bias strongly suspected^d^ | 314 | 309 | - | MD **1.89 lower** (3.74 lower to 0.04 lower) | ⨁◯◯◯ Very low |
| **Clinical success ‒ Resectable** | | | | | | | | | | | |
| 2 | Observational studies | Very serious^a^ | Very serious^b^ | Not serious | Very serious^c^ | None | 50/63 (79.4%) | 62/72 (86.1%) | Not estimable | **40 fewer per 1.000** (from 400 fewer to 320 more) | ⨁◯◯◯ Very low |
| **Technical success ‒ Resectable** | | | | | | | | | | | |
| 6 | Observational studies | Very serious^a^ | Serious^f^ | Not serious | Serious^e^ | None | 234/277 (84.5%) | 293/359 (81.6%) | Not estimable | **60 more per 1.000** (from 10 more to 120 more) | ⨁◯◯◯ Very low |
| **Crossover ‒ Resectable** | | | | | | | | | | | |
| 4 | Observational studies | Very serious^a^ | Very serious^b^ | Not serious | Not serious | None | 5/129 (3.9%) | 71/252 (28.2%) | Not estimable | **290 fewer per 1.000** (from 510 fewer to 70 fewer) | ⨁◯◯◯ Very low |
| **Post-drainage complications ‒ Resectable** | | | | | | | | | | | |
| 8 | Observational studies | Very serious^a^ | Very serious^b^ | Not serious | Not serious | Publication bias strongly suspected^d^ | 107/375 (28.5%) | 273/514 (53.1%) | Not estimable | **200 fewer per 1.000** (from 330 fewer to 60 fewer) | ⨁◯◯◯ Very low |
| **Cholangitis ‒ Resectable** | | | | | | | | | | | |
| 7 | Observational studies | Very serious^a^ | Very serious^b^ | Not serious | Not serious | Publication bias strongly suspected^d^ | 75/339 (22.1%) | 154/469 (32.8%) | Not estimable | **100 fewer per 1.000** (from 230 fewer to 20 more) | ⨁◯◯◯ Very low |
| **Pancreatitis ‒ Resectable** | | | | | | | | | | | |
| 8 | Observational studies | Very serious^a^ | Very serious^b^ | Not serious | Not serious | Publication bias strongly suspected^d^ | 3/375 (0.8%) | 57/514 (11.1%) | Not estimable | **100 fewer per 1.000** (from 160 fewer to 50 fewer) | ⨁◯◯◯ Very low |
| **Bleeding ‒ Resectable** | | | | | | | | | | | |
| 6 | Observational studies | Very serious^a^ | Not serious | Not serious | Very serious^c^ | Publication bias strongly suspected^d^ | 6/272 (2.2%) | 6/395 (1.5%) | Not estimable | **0 fewer per 1.000** (from 30 fewer to 30 more) | ⨁◯◯◯ Very low |
| **Serious complications ‒ Resectable** | | | | | | | | | | | |
| 7 | Observational studies | Very serious^a^ | Very serious^b^ | Not serious | Very serious^c^ | Publication bias strongly suspected^d^ | 51/339 (15.0%) | 77/469 (16.4%) | Not estimable | **10 more per 1.000** (from 60 fewer to 80 more) | ⨁◯◯◯ Very low |

CI, Confidence Interval; MD, Mean Difference.

Explanations: ^a^According to ROBINS-I; ^b^Heterogeneity >60%; ^c^ Very large gap in NNT; ^d^ Outlier; ^e^ Large gap in NNT; ^f^ Heterogeneity >50% and <60%; ^g^> 2 SD.

**Supplementary Material – Appendix 2** Quality of evidence by GRADE for palliative PCCA.

| **Certainty assessment** | | | | | | | **Nº of patients** | | **Effect** | | **Certainty** |
| --- | --- | --- | --- | --- | --- | --- | --- | --- | --- | --- | --- |
| **Nº of studies** | **Study design** | **Risk of bias** | **Inconsistency** | **Indirectness** | **Imprecision** | **Other considerations** | **PTBD** | **EBD** | **Relative (95% CI)** | **Absolute (95% CI)** |  |
| **Clinical success ‒ Palliative** | | | | | | | | | | | |
| 4 | Observational studies | Very serious^a^ | Not serious | Not serious | Not serious | None | 197/229 (86.0%) | 154/260 (59.2%) | Not estimable | **190 more per 1.000** (from 110 more to 270 more) | ⨁◯◯◯ Very low |
| **Technical success ‒ Palliative** | | | | | | | | | | | |
| 2 | Observational studies | Very serious^a^ | Very serious^b^ | Not serious | Not serious | None | 138/139 (99.3%) | 240/260 (92.3%) | Not estimable | **100 more per 1.000** (from 220 fewer to 420 more) | ⨁◯◯◯ Very low |
| **Crossover ‒ Palliative** | | | | | | | | | | | |
| 2 | Observational studies | Very serious^a^ | Very serious^b^ | Not serious | Not serious | None | 1/81 (1.2%) | 9/107 (8.4%) | Not estimable | **120 fewer per 1.000** (from 360 fewer to 110 more) | ⨁◯◯◯ Very low |
| **Post-drainage complications ‒ Palliative** | | | | | | | | | | | |
| 6 | Observational studies | Very serious^a^ | Very serious^b^ | Not serious | Serious^e^ | Publication bias strongly suspected^d^ | 94/310 (30.3%) | 119/324 (36.7%) | Not estimable | **80 fewer per 1.000** (from 210 fewer to 60 more) | ⨁◯◯◯ Very low |
| **Cholangitis ‒ Palliative** | | | | | | | | | | | |
| 6 | Observational studies | Very serious^a^ | Not serious | Not serious | Serious^e^ | Publication bias strongly suspected^d^ | 61/310 (19.7%) | 95/324 (29.3%) | Not estimable | **80 fewer per 1.000** (from 150 fewer to 10 fewer) | ⨁◯◯◯ Very low |
| **Pancreatitis ‒ Palliative** | | | | | | | | | | | |
| 5 | Observational studies | Very serious^a^ | Not serious | Not serious | Very serious^c^ | Publication bias strongly suspected^d^ | 9/262 (3.4%) | 10/227 (4.4%) | Not estimable | **10 fewer per 1.000** (from 50 fewer to 30 more) | ⨁◯◯◯ Very low |
| **Bleeding ‒ Palliative** | | | | | | | | | | | |
| 6 | Observational studies | Very serious^a^ | Not serious | Not serious | Very serious^c^ | Publication bias strongly suspected^d^ | 19/310 (6.1%) | 6/324 (1.9%) | Not estimable | **30 more per 1.000** (from 0 fewer to 60 more) | ⨁◯◯◯ Very low |
| **Serious complications ‒ Palliative** | | | | | | | | | | | |
| 6 | Observational studies | Very serious^a^ | Not serious | Not serious | Very serious^c^ | Publication bias strongly suspected^d^ | 21/310 (6.8%) | 11/324 (3.4%) | Not estimable | **10 more per 1.000** (from 20 fewer to 50 more) | ⨁◯◯◯ Very low |

CI, Confidence Interval; MD, Mean Difference.

Explanations: ^a^According to ROBINS-I; ^b^Heterogeneity >60%; ^c^ Very large gap in NNT; ^d^ Outlier; ^e^ Large gap in NNT.
